# Gut Microbiota and Lifestyle Interventions in NAFLD

**DOI:** 10.3390/ijms17040447

**Published:** 2016-03-25

**Authors:** David Houghton, Christopher J. Stewart, Christopher P. Day, Michael Trenell

**Affiliations:** 1 Institute of Cellular Medicine, Newcastle University, Newcastle upon Tyne NE4 6BE, UK; chris.day@newcastle.ac.uk; 2 Alkek Center for Metagenomics and Microbiome Research, Department of Molecular Virology and Microbiology, Baylor College of Medicine, Houston, TX 77030, USA; Christopher.Stewart@bcm.edu; 3 Liver Unit, Newcastle upon Tyne Hospitals NHS Trust, Freeman Hospital, Newcastle upon Tyne NE7 7DN, UK

**Keywords:** NAFLD, gut microbiota, lifestyle, diet and exercise

## Abstract

The human digestive system harbors a diverse and complex community of microorganisms that work in a symbiotic fashion with the host, contributing to metabolism, immune response and intestinal architecture. However, disruption of a stable and diverse community, termed “dysbiosis”, has been shown to have a profound impact upon health and disease. Emerging data demonstrate dysbiosis of the gut microbiota to be linked with non-alcoholic fatty liver disease (NAFLD). Although the exact mechanism(s) remain unknown, inflammation, damage to the intestinal membrane, and translocation of bacteria have all been suggested. Lifestyle intervention is undoubtedly effective at improving NAFLD, however, not all patients respond to these in the same manner. Furthermore, studies investigating the effects of lifestyle interventions on the gut microbiota in NAFLD patients are lacking. A deeper understanding of how different aspects of lifestyle (diet/nutrition/exercise) affect the host–microbiome interaction may allow for a more tailored approach to lifestyle intervention. With gut microbiota representing a key element of personalized medicine and nutrition, we review the effects of lifestyle interventions (diet and physical activity/exercise) on gut microbiota and how this impacts upon NAFLD prognosis.

## 1. Non-Alcoholic Fatty Liver Disease (NAFLD)

Non-alcoholic fatty liver disease (NAFLD) represents a spectrum of liver disease including simple steatosis, non-alcoholic steatohepatitis (NASH), fibrosis and cirrhosis, in the absence of excessive alcohol consumption [1]. NAFLD is the leading aetiology of liver disease [2], although factors leading to the development of NAFLD and progression to more advanced liver disease are poorly understood [3]. NAFLD is strongly associated with metabolic syndrome and its features including insulin resistance, obesity, hyperlipidemia, low high density lipoproteins (HDL), and hypertension and is considered the hepatic manifestation of the metabolic syndrome [4].

The incidence of NAFLD is closely associated with dietary intake and lack of physical activity, which typically manifests in obesity [5]. NAFLD is further accompanied by excess risk of type 2 diabetes mellitus (T2DM) and cardiovascular disease (CVD) [6]. The multifactorial aetiology of NAFLD is determined by both the patient’s genetics and the environmental factors to which they are exposed, which may account for the substantial inter-patient variability common to the disease [7]. Although genetic polymorphisms have been attributed to account for a small portion of the patient inter-variability, there are additional contributing factors that have also been identified, spanning epigenetics, hormones, nutrition and physical inactivity [5,7]. Despite advances in NAFLD pathology, the reasons for the large inter-patient variability in progression remains incompletely understood. Consequently, a potential new diagnostic and therapeutic target receiving considerable attention is the collection of microorganisms that reside the gastrointestinal (GI) system. Despite humans being >99% identical genetically, the collection of bacteria, fungi, archea, virus, and phage are hugely diverse and highly individual from one person to the next. Termed the gut microbiota, bacteria in the gut alone accounts for around 70% of the total bacteria in the body and include 500–1000 different bacterial species [8,9,10,11].

Bacterial evolutionary linages are represented by phylogenetic trees, demonstrating the relatedness of bacteria to one another, classified from life, domain, kingdom, phylum, class, order, family, genus and finally species. The majority of research into gut microbiota has focused on phylum (Firmicutes, Bacteroidetes, *etc.*), genus (*Bacteroides*, *Lactobacillus*, *etc.*), and species (*Roseburia* spp. and *Eubacterium* spp.).

## 2. Gut Microbiota

Historical evidence spanning eight decades has demonstrated a link between the bacteria in the GI system and the liver, present from early fetal life and throughout life [8,12]. The gut microbiota in a healthy individual has been shown to be stable, absent of clinical manipulation (e.g., antibiotics), provided that a healthy diet and physical activity, combined with a healthy lifestyle (e.g., limited alcohol, not smoking, *etc.*) are maintained [13,14,15,16,17,18]. A healthy balance of bacteria in the GI system ensures that the gut microbiota works in a symbiotic nature with the host and its functions include maintaining a supply of essential nutrients, metabolism, immune response and intestinal architecture [19]. However, a change in the diversity leading to a reduced abundance of beneficial bacteria, with increased prevalence of potentially pathogenic bacteria can occur, which has been termed “dysbiosis” [17]. Dysbiosis of the gut microbiota has been associated with many disease states from early infancy [20], through childhood [21] and into adulthood [18]. Thus, manipulation of the gut microbiome to ensure a non-dysbiotic state offers attractive therapeutic for a range of conditions and overall health status.

Synonymous to NAFLD, inter-patient variability of the gut microbiota is well recognized, with each individual harboring a unique collection of microorganisms from the thousands that can potentially colonize, primarily from the phyla Firmicutes, Bacteroides and Actinobacteria [15,22]. Until recently, the majority of research published focused on these phyla, specifically the Firmicutes and Bacteroides, which are dominant in the gut microbiota from year three of life. However, recent advances in the throughput and affordability of deoxyribonucleic acid (DNA) sequencing technologies and associated bioinformatics [23] has facilitated an increased understanding of the pathophysiology of a number of diseases and adverse health conditions including obesity, metabolic syndrome, diabetes and cardiovascular disease [18,24,25,26], all of which are closely associated with NAFLD [4].

## 3. Gut Microbiota and NAFLD

NAFLD is a complex disease and with advances in the pathology of the disease new pharmacotherapy treatments are being developed [27,28]. However, lifestyle interventions accompanied by weight loss of between 5% and 10% remain the cornerstone of treatment [27,29]. The effectiveness of lifestyle changes are unprecedented with improvements in metabolic control and liver histology, and when accompanied by greater than 10% weight loss NASH resolution, fibrosis regression and reductions NAFLD activity score [30,31]. However, the difficulty in implementing and maintaining these lifestyle interventions in clinical practice in NAFLD is well documented, with randomized long-term studies lacking [32,33].

**Figure 1:**
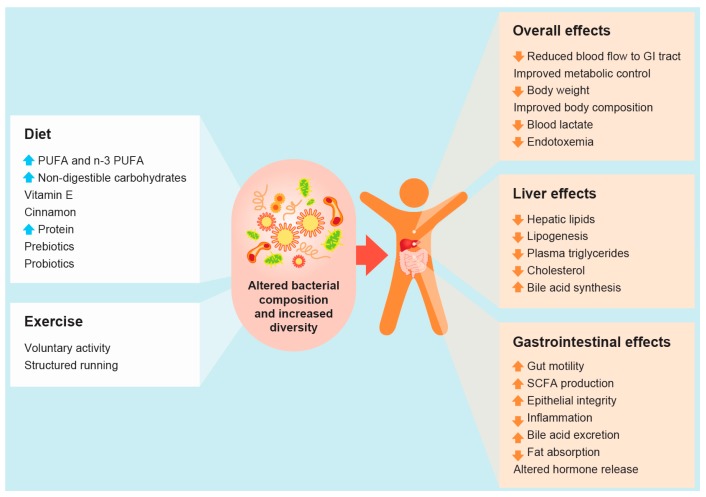
Impact of lifestyle interventions on gut microbiota and non-alcoholic fatty liver disease (NAFLD) and its risk factors (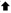 and 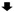 arrows denote increase or decrease in variables, respectively).

Due to the intimate relationship between liver and GI tract, it is unsurprising that gut microbiota dysbiosis has been linked with hepatic fat accumulation, and all stages of NAFLD in both animals and humans [7,34,35,36,37,38,39,40,41]. Although the exact mechanism linking the gut microbiota with NAFLD development and progression remains unknown, potential explanations include bacterial overgrowth, gut leakiness, increased endotoxemia absorption, and inflammation [3,36,42,43,44,45]. The increased knowledge of the gut microbiota in recent years has enhanced the understanding of the metabolic and immunological potential and microbial–host interactions, primarily in gut, but also in the liver and other organs. The role and identity of microbial produced metabolites and their direct function locally in the gut and also at other body sites remains unknown. However, increasing evidence suggests the gut microbiota as a genuine target for therapeutic interventions in the management of NAFLD (Figure 1) [8,19].

This review provides an overview of gut microbiota and its relationship with NAFLD by reviewing published data on how diet, nutrition and exercise modulate the gut microbiota and the liver. The purpose of this review is to assess the impact that lifestyle interventions (excluding pharmaceutical and surgical) have on gut microbiota and how this may interact with NAFLD development and progression.

## 4. Lifestyle Interventions in NAFLD

As the incidence of NAFLD increases [1], the individual and societal burden of its management weigh heavily on health care systems throughout the world, and the need for treatments to combat this is crucial [46]. Although the understanding of NAFLD has increased considerably in the last 20 years, the exact cause of why some people develop more severe forms of NAFLD is not fully understood. The development of NAFLD results from two key factors: (1) greater calories consumed compared to those expended; and (2) genetic susceptibility. Although genetic susceptibility cannot be altered (excluding epigenetic changes), calories consumed and expended can be modified, and has led to a large body of research undertaken investigating the impact of various lifestyle modification interventions [27,29]. Lifestyle interventions that lead to a reduction in weight and/or an increase in physical activity/exercise have consistently been shown to reduce hepatic lipids, improve glucose control and insulin sensitivity [29], and more recently improve liver histology [30,31]. The control of calories consumed *vs.* those expended may incorporate a number of interventions including exercise/physical activity independent of weight loss [47,48,49], diet modification [50,51,52] or diet and exercise/physical activity [30,31,53,54]. However, why some patients respond to interventions, and others do not is unknown. Modulation of the gut microbiota through the various lifestyle modifications discussed here may provide an insight into the inter-patient variability observed in NAFLD, and improve the number of people who may respond to specific lifestyle interventions to treat NAFLD.

## 5. Diet and Gut Microbiota

Exposure to environmental factors plays a significant role in the pathophysiology of NAFLD [6], particular dietary intake [55]. A regular healthy balanced diet has been shown to maintain a stable and healthy gut microbiota and reduce the risk of numerous diseases [56,57]. Recent evidence has emphasized the importance of calorie excess, in contrast to macronutrient content, as a major contributor to weight gain [58]. This is particularly important given the highly calorific content of the Western diet (high in fat and carbohydrates), which is associated with an altered gut microbiota and increased risk of developing obesity and NAFLD [35,46,59]. Although there are some conflicting findings, a strong association has been reported between obesity and changes in the gut microbiota, which may be responsible for enhanced energy harvest, weight gain and metabolic syndrome [60,61,62]. The link between the diet and the composition and function of the gut microbiota is unsurprising given that dietary components provide nutrients for bacteria, which then produce metabolites involved in energy balance, metabolism, immune response and the pathophysiology of NAFLD [63,64,65]. Indeed, bacteria in the gut are responsible for the digestion and production of many essential vitamins and minerals. The link between diet, gut microbiota, and health has been elegantly shown in animal models. Animals that were switched from low fat/fiber rich plant diets, to high fat/high sugar diets had significant increases in Bacilli and Erysipelotrichi from the Firmicutes phylum, which were associated with a significant decrease in the abundance of members of the Bacteroidetes phylum [66]. Furthermore, the role of the gut microbiome alone in causing obesity, independent of diet, was first demonstrated by Ley *et al.* [67] who showed mice transplanted an “obese microbiota” would have significantly greater weight gain than mice transplanted with a “lean microbiota”. The substantial impact of the diet has also been shown in humans, where a rural African diet (high in fiber and vegetables) had a higher relative abundance of Bacteroidetes and a lower relative abundance of Firmicutes compared with the urban European diet (high in fat and sugar, low in fiber and vegetables). Even more interesting was that the samples from Africa had two bacterial species (*Prevottela* and *Xylanibacter*) that were not detectable in the European samples. Further evidence has been reported when comparing a control diet *vs.* diets high in non-digestible carbohydrates, where the authors reported that the non-digestible carbohydrates produce significant changes in the composition of the gut microbiota within a number of days [62].

Although there are contrasting results in the specific bacterial taxa that are modulated through the diet, the key message is that the diet is able to have a direct and long-term impact on the gut microbiota composition and function, which has a profound implication for health. Any modulation of the diet, such as an increase in non-digestible carbohydrates and/or weight loss has the potential to alter the gut microbiota and potentially disease phenotype, such as NAFLD. This approach of modulating the gut microbiota by modifying the dietary components (fats, proteins and carbohydrates), probiotics (living microorganisms that provide health effects on the host), and prebiotics (ingredients that are selectively fermented and modulate the changes in both the composition and activity of the gut microbiota) has been established for some time, although the links between specific bacteria with disease and mechanisms are often lacking [68]. This paper will now report the impact that macronutrients (fats, proteins and carbohydrates), probiotics and prebiotics manipulation has on the gut microbiota and the NAFLD phenotype.

## 6. Fat

Although the exact pathophysiology of NAFLD is unknown, the accumulation of lipids in the liver is a key pre-requisite for development and progression [69]. The cause of lipid accumulation in the liver is complex, but has been linked with an influx of fatty acids from fat depots, *de-novo* lipogenesis, and excess dietary fat intake, leading to steatosis. Increased fat intake is a common finding in NAFLD patients [70,71], thus regulation of fat intake has been highlighted as potential target for therapeutic intervention to reduce hepatic lipids [72]. Contrasting results have been reported in human studies that have used a high fat diet to increase hepatic lipid content [73,74], whereas others have reported no effect of a high fat diet on hepatic lipids [75,76]. The lack of consistency is likely to be due to the duration of the studies (10 days–3 weeks) and the various forms of fat used (saturated, polyunsaturated (PUFA) and mono-unsaturated (MUFA)). Furthermore, in a regular Western style diet the high fat content is normally supplemented by high carbohydrates and therefore it may be the combination of fat and carbohydrates that stimulate the development and progression of NAFLD [77]. The Western diet associated with NAFLD has also been associated with gut microbiota dysbiosis, which represents a potential source for the inter-patient variability observed in NAFLD, and progression from simple steatosis to NASH [36,78].

Although the exact mechanisms of how high fat diets lead to the development of NAFLD through gut microbiota modulation are unknown, research has predominantly focused on gut barrier function [77], leaky gut, endotoxemia, gut derived toxins and inflammation [45,79]. Despite the links between high fat diets, NAFLD, and the gut microbiota, there is a need to identify specific microbial changes that may be causative, which would highlight potential targets for diagnosis and treatment. The majority of studies investigating the impact that a high fat diet can have on the gut microbiota have been based around changes in the Firmicutes:Bacteroidetes ratio. This was firstly shown by Turnbaugh, *et al.* [80] in germ free mice that were fed a high fat diet and failed to develop obesity. Once inoculated with the microbiota from a mouse fed a high fat diet, the mice had increased weight, hepatic lipogenesis, fat deposition and insulin resistance, which was associated with an increase in Firmicutes and a subsequent decrease in Bacteroidetes [80]. This has subsequently been supported by decreases in *Eubacterium rectale*, *Blautia coccoides*, *Bifidobacteria* sp. and *Bacteroides* sp. [81,82,83,84]. Although these studies have identified that the gut microbiota is modulated with a high fat diet, the changes reported are limiting in their specificity (phylum changes rather than species), and the exact mechanism(s) linking these changes with NAFLD require further investigation.

There have been a number of mechanisms that have been identified to play a role in gut microbiota dysbiois associated with a high fat diet and the development of NAFLD including gut barrier dysfunction and translocation of microbes from the gut. Increased endotoxemia and inflammation in human [85,86,87] and animal studies [81,88,89,90] further suggests insulin resistance to be a key to the development of NAFLD and NASH [90,91]. High fat diets have also been shown to modulate the levels of Gammaproteobacteria and Erysipelotrichi, which have been shown to lead to choline deficiency, liver fat accumulation and NASH [65,92,93,94]. In addition, the ability of high fat diets to alter the gut microbiota and subsequently bile acids metabolism and synthesis by alleviating farnesoid X receptor (FXR) [95,96,97]. Although not exhaustive, the mechanisms discussed here have all been shown to have a direct impact upon the liver, therefore modulation of the gut microbiota in the presence of a high fat diet may offer the potential to reduce the risk and development of NAFLD.

The obvious treatment may be to put NAFLD patients on a low fat diet which have been shown to be effective in weight loss, reduce liver fat, improve metabolic control and modulate the gut microbiota [10,30,98]. However, managing and maintaining such diets can be difficult [32,33]. A number of alternative options for patients who may struggle with converting to a low fat diet include changing the form of fat consumed and increasing non-digestible carbohydrates. MUFA, PUFA and *n*-3 PUFA have been incorporated into dietary studies and shown to restore aspects of the high fat gut microbiota dysbiosis, including changes in Clostridia, Enterobacteriales, *Bifidobacterium* and *Lactobacillus* *casei* (Table 1) [99,100]. Although these studies do not report how these changes link with NAFLD, potential explanations may include reduced gut leakiness and inflammation, although these were not confirmed. In human studies, increases in MUFA, PUFA and *n*-3 PUFA have been shown to reduce hepatic lipid content and improve metabolic control in NAFLD patients [73,100], potentially due to increase fatty acid oxidation, redistribution of fatty acids and down regulation of gene expression of sterol regulatory element binding protein 1 (SREBP1-c) and factor for apoptosis (FAS). It is important to emphasize that the changes reported here did not influence weight, therefore suggesting the changes in the gut microbiota and hepatic lipids are diet driven rather than weight loss.

The use of non-digestible carbohydrates has been researched for a number of years and shown to be an effective treatment for increasing satiety, reducing blood glucose, insulin resistance, fat digestion and inducing weight loss [101]. Furthermore, non-digestible carbohydrates are effective in modulating gut microbiota and maintaining a healthy GI system [64]. However, the impact upon the gut microbiota in the presence of a high fat diet and mediators of NAFLD are lacking. Arabinoxylan and chitin-glucan have been shown to be effective at modulating the gut microbiota by increasing Bifidobacteria and restoring the abundance of *Bacteroides*-*Prevotella* spp., *Roseburia* spp., and *Clostridium* cluster fourteen a (XIVa) that were reduced following a high fat diet (Table 1). These changes in the gut microbiota were also supported by reductions in body fat, hepatic lipids, serum and hepatic cholesterol and insulin resistance, independent of calories consumed [102,103]. There is also evidence that, in the presence of high fat diets, chitosan and arabinoxylan are able to increase fat, bile acids and cholesterol in the feces. These studies suggest that non-digestible carbohydrates are able to modulate the gut microbiota, even in the presence of a high fat diet, potentially by binding to fat/cholesterol or inhibiting pancreatic lipase [101,104,105].

Although a low fat diet may be preferable for patients with NAFLD, such a considerable change from an established lifestyle will be difficult for patients with NAFLD to incorporate. It is also important to recognize that, in general, a reduction in fat intake is typically accompanied by an increase in carbohydrate content. The data here suggest that changing the type of fat ingested and incorporating a larger proportion of non-digestible carbohydrates into the diet may be effective modulating the gut microbiota, reducing hepatic lipids and ameliorating risk factors associated with NAFLD. However, further work is required to assess the impact of diet on the gut microbiota specifically and further human intervention studies in patients with NAFLD are required to assess this.

## 7. Carbohydrates

Carbohydrates provide a crucial energy source for the host and gut microbiota [25]. Carbohydrate fermentation, specifically non-digestible carbohydrates, is a core activity of the human gut microbiota, driving the energy and carbon economy of the colon [106]. The move towards the Western style diet, which is high in processed carbohydrates and low in non-digestible carbohydrates, has been attributed to the rise and prevalence of obesity and NAFLD in these demographics [8,65,107]. This was recently confirmed in a meta-analysis where fructose was linked with poor liver health, although this was confounded by excessive energy intake, which is likely to be due to high fructose intake [108].

Excessive intake of calories in NAFLD is associated with sugar intake, with fructose being identified as having a crucial role to play (potentially due to altered hormone release). With regard to NAFLD, excess fructose, which is primarily metabolized in the liver, is linked to elevated steatosis [109,110,111]. Fructose has also been suggested to be a key driver in alteration of gut microbiota, potentially causing dysbiosis, as well as increased intestinal permeability and endotoxins in portal blood [112,113]. Notably, such factors have been previously reported in NAFLD [114]. Increased endotoxins and inflammatory cytokines have been identified to be part of the multiple hits hypothesis that exposes the liver to inflammation and injury [91]. Furthermore, endotoxemia is also linked with activation of Kupffer cells through toll like receptor dependent mechanisms, weight gain, poor metabolic control and increased plasma triglycerides, hepatic lipogenic enzymes and hepatic steatosis [111,113,115,116,117,118].

Replacing non-digestible carbohydrates with simple carbohydrates, such as fructose, will alter the substrate made available to the gut microbiota and ultimately affect the metabolic outputs and the microbial composition [67,106,119]. Numerous studies have reported that reducing non-digestible carbohydrates in the diet significantly reduces the levels of *Roseburia* spp. and *Eubacterium rectale* subgroup of cluster XIVa from the Firmicutes phylum and bifidobacteria from the Actinobacteria phylum [98,120]. More recently, the same group have also shown that increasing the levels of non-digestible carbohydrates can increase levels of *Ruminococcus bromii* (phylum:Firmicutes), however, these changes were dependent on the individuals initial microbiota profile [62]. These changes reflect the impact that non-digestible carbohydrates have on gut microbiota and subsequent health implications, although studies linking carbohydrates intake, such as non-digestible carbohydrates and fructose, with specific bacterial changes in NAFLD are lacking.

There are few studies investigating the effects of high carbohydrate intake similar to the Western diet on the gut microbiota composition. Ferrere, *et al.* [121] reported increased relative abundance of the class Erysipelotrichi (phylum:Firmicutes) following high fructose diet. Turnbaugh, Backhed, Fulton and Gordon [84] also reported that a high carbohydrate diet was able to increase the relative abundance of bacteria from the class Mollicutes (phylum:Firmicutes) and enrich genes that encode fructose metabolism, but reduce genes required for starch and sucrose metabolism. The authors suggested that the increase in Mollicutes might reduce microbial diversity, including a reduction in the relative abundance of the genus *Bacteroides* (phylum:Bacteroidetes), which is associated with poor health [61]. However, in humans Boursier, Mueller, Barret, Machado, Fizanne, Araujo-Perez, Guy, Seed, Rawls, David, Hunault, Oberti, Cales and Diehl [7] reported increased levels of *Bacteroides* in NASH patients compared to controls, which suggests that the data which we extrapolate from animal models require validation in human populations. Further studies should ascertain the effect of high fructose diets on specific bacteria to potentially identify targets for treatment.

The cornerstone of NAFLD treatment is weight loss, through diet and/or physical activity/exercise, which is effective in improving both liver histology [30] and modulating the gut microbiota [10,31]. In recent years a reduction in calories in the form of carbohydrates has been prevalent in many fad diets. Although initially effective for weight loss, such diets also have a substantial impact upon the gut microbiota and short chain fatty acids (SCFA) [98]. SCFA contribute around 10% of our daily energy requirements and provide a hospitable environment for cross feeding between microbial communities [108]. Specifically, a reduction in butyrate and butyrate producing bacteria has been shown in such diets, which may have detrimental effects on the GI structure and immune response [98,106,120]. An alternative would be to increase the amount of non-digestible carbohydrates consumed, which has been shown to be effective in maintaining a healthy gut microbiota [67] and ameliorating obesity and insulin resistance, which appear to be necessary for the development of NAFLD [122,123]. Furthermore, SCFA, including butyrate have been shown to contribute towards maintaining epithelial integrity, gut motility, hormone secretion, reducing appetite and inflammation [106], all of which are associated with NAFLD [122,123]. Increased intake of non-digestible carbohydrates has also been shown to improve glucose uptake, adipokine profile, and alter colonic fermentation, although the latter was only confirmed with breath tests [124]. Oligofructose specifically has also been shown to induce weight loss, reduce calories consumed and improved glucose uptake [125]. The authors also reported reductions in grehlin and increased peptide YY response, which have both been associated with changes in the gut microbiota following dietary intervention [126,127].

Further treatments to combat the detrimental effects of a high carbohydrate diet may involve increased protein intake, Vitamin E, and cinnamon [128,129,130,131]. All of which have been shown to be effective in reducing weight gain, body fat, adipocyte size, insulin resistance and hepatic steatosis. Although these all show promise, there are currently no data on whether these may be able to modulate the gut microbiota in NAFLD patients and should be explored further. Increased intake of carbohydrates, specifically fructose, is undoubtedly linked with NAFLD, due to either metabolism in the liver or through increasing calories consumed. Replacement of these simple carbohydrates with non-digestible carbohydrates provides potential to have a direct impact upon gut microbiota dysbiosis and have a positive effect on mediators of NAFLD.

## 8. Protein

Like carbohydrate, an increase in protein in the diet at the expense of carbohydrates and fat had been utilized in a number of fad diets to facilitate weight loss [132]. However, the effect that protein may have on the gut microbiota in humans, and specifically NAFLD are lacking. Furthermore, the small number of studies investigating the effects of high protein diets have predominantly focused on the products produced during fermentation [64]. This is surprising given the amount of protein that reaches the colon in a healthy diet (12–18 g), which would be expected to rise in a high protein diet [133], and provide nutrients for bacterial proliferation. Although an essential macronutrient, excess protein has been linked with potentially damaging effects on the gut microbiota and intestinal structure through toxic substances produced [64,133,134,135]. The small number of studies that have reported the impact that high protein diets have on the gut microbiota have reported high levels of *Clostridium* spp. and *Bacteroides* spp., with concurrent reductions in *Bifidobacterium* spp., *Roseburia* spp., and *Eubacterium* spp. [136,137]. The reductions in *Bifidobacterium* spp., *Roseburia* spp., and *Eubacterium* spp. bacterial species are associated with butyrate production, endotoxemia, mucus barrier function, and insulin sensitivity [81,136]. This suggests that the decreases in these bacteria may be detrimental to health and increase the risk of NAFLD [35,36,37,40,138,139].

Various animal models have been used to investigate the impact of protein on the gut microbiome with the main findings summarized in Table 2. In cats the authors utilized a high protein/low carbohydrate and a moderate protein and carbohydrate diet for eight weeks [140]. Ordination analysis of this data demonstrated that increases in the abundance of genre *Clostridium*, *Faecalibacterium*, *Ruminococcus*, *Blautia* and *Eubacterium* were clustered with plasma triglycerides. Contrastingly, piglets fed a high protein diet showed little microbial change, except reductions in *Faecalibacterium prausnitzii*, but were shown to have a higher increase in colonic permeability and higher cytokine secretion [141]. Whether this small change in bacteria can be directly attributed to the bacterial changes remains to be seen. However, the differing animal models and protein sources may account for the difference reported here. Further animal data from chickens and rats fed diets high in protein showed reductions in hepatic lipids and adipose tissue [142,143,144]. More recently, high protein diets have been shown to be effective for reducing hepatic lipids, blood lipids, body fat, CVD risk and improve insulin sensitivity and anti-oxidative potential [132,145,146,147,148,149]. Potential explanations for these results have been linked with increased satiety, increased energy expenditure, reduced hepatic lipid oxidation, cell death, hormone release in the GI system and bile acid metabolism [126,127,150], all of which are associated with the pathophysiology of NAFLD [123].

Although there has been no direct link between the gut microbiota, NAFLD, and a high protein diet, there is evidence that an excess of 36 g/day of protein was identified as a risk factor for increasing the risk of NAFLD [111]. Furthermore, in T2DM an increase in protein intake resulted in reduced insulin sensitivity, increased gluconeogenesis and increase glucagon [151,152].

Overall, there are contrasting evidence on whether diets high in protein may be an effective treatment for gut microbiota modulation and NAFLD management. Discrepancies are likely due to differing study designs, including the source of protein (animal *vs.* plant protein), varying manipulations of the diets (protein to carbohydrate and fat ratios) populations sampled (Healthy, NAFLD, T2DM, obese, *etc.*) and study duration. The optimal benefits of diets high in protein to modulate the gut microbiota and aid with NAFLD management strategies should be explored further.

## 9. Prebiotics and Probiotics

Given the connection between the gut microbiota, diet, and health [65,84], coupled to issues with sustained dietary modification, an alternative approach utilizes pre- and probiotics to indirectly or directly confer beneficial colonizers of the GI, respectively [153]. Although there are various definitions, prebiotics is most commonly referred to as ingredients that are selectively fermented and modulate the changes in both the composition and activity of the gut microbiota [68,154]. Probiotics are different in that they are living (viable) microorganisms which have the ability to provide health effects on the host when provided in adequate amounts, similar to the bacteria that are already present [155].

## 10. Prebiotics

Fructans are the most extensively studied prebiotics and have been linked with modulation of the gut microbiota (summarized in Table 3), resulting in positive health benefits. In animal models, the administration of prebiotics increased 18 potentially beneficial species, notably *Bifidobacterium* spp. (Phylum: Actinobacteria) and Bacteroidetes [156,157,158]. Changes in gut microbiota are also associated with appetite regulation, improved glucose tolerance, increased satiety, reduced ghrelin, plasma triglycerides, oxidative stress and inflammation and calories consumed [82,158,159,160].

Further animal data have demonstrated that prebiotics are able to reduce hepatic lipids, cholesterol, plasma triglycerides and increase the SCFA propionate [161,162,163]. Daubioul, Rousseau, Demeure, Gallez, Taper, Declerck and Delzenne [161] suggested that the improved lipid profile and hepatic lipids were due to changes in the gut microbiota, which ultimately altered metabolites of fermentation. Alterations in the acetate:propionate ratio has been shown to reduce lipogenesis and account for the reductions in hepatic lipids. Although the authors suggest that the changes in SCFA are due to modulation of the gut microbiota, the authors failed to measure specific bacteria, focusing rather on the metabolites. It is intriguing that increased SCFA production, specifically butyrate and propionate, protect against diet-induced obesity in mice [164]. However, such studies underscore the need to understand the complex interplay between microbial–host interaction in gut and to which extent the bacterial community is causing the phenotypes observed.

In human cohorts, where systematic study is challenging, studies elucidating the exact effects and mechanisms of prebiotics on the gut microbiota and resulting microbial–host interaction are lacking. Studies in healthy and T2DM patients have provided similar results, reporting increased satiety and reduced ghrelin, body weight, glucose, and inflammation [125,157,165,166]. There is a need for studies investigating the effects of prebiotics in NAFLD. In a small pilot study in biopsy confirmed NASH patients, Daubioul, *et al.* [167] reported that prebiotics had a positive impact on liver aminotransferases and insulin, but no effects on plasma triglycerides. A recent study reported that prebiotics were effective at significantly reducing inflammatory cytokines, liver aminotransferases, insulin sensitivity and steatosis in NASH patients [168]. Notably, the study compares lifestyle alone with lifestyle and prebiotics, thus it is difficult to ascertain how much affect the prebiotics with no other lifestyle interventions alone would have. As with existing animal data, such studies lack analysis of the gut microbiota and thus relating potential mechanisms to changes in the gut microbiota is not possible.

A recent meta-analysis analyzing 26 randomized controlled trials concluded that prebiotics were effective in increasing satiety and improving insulin sensitivity [169]. While specific effects of prebiotics on the gut microbiota remain poorly understood, the majority of studies have reported increases in Bifidobacteria [170,171,172,173]. Dewulf, *et al.* [174] also reported an increase in Bifidobacteria, as well as increased levels of *Faecalibacterium prausnitzii* and reductions in *Bacteroides intestinalis*, *Bacteroides vulgatus* and *Propionibacterium*, which they associated with endotoxemia, although they failed to report any changes in plasma endotoxemia. Although these studies demonstrate promise that prebiotics may be used as a potential treatment for NAFLD, further work is required to investigate additional overall changes in the gut microbiome. Rapid advances in NGS and other ‘omic technologies offer promise for more systematic understanding of entire treatment mechanisms. In addition, there are currently no studies that have reported the effects of prebiotics on hepatic lipids or liver histology, and evidence linking specific bacteria with the pathophysiology of NAFLD is lacking.

## 11. Probiotics

Probiotics have been suggested as a potential treatment for patients with NAFLD, due to their apparent ability to modulate the gut microbiota (Table 4) and impact upon metabolic control, inflammation, lipid profile and intestinal permeability [155,175], and have been systematically reviewed in detail elsewhere [176]. However, the exact mechanisms of how probiotics are able to do this are not fully understood. Although not exhaustive, proposed mechanisms include direct microbe-to-microbe interaction and competition with pathogenic bacteria potentially leading to eradication and a healthy balance of gut microbiota [62,177]. Furthermore, probiotics have been shown to be effective at improving epithelial barrier integrity [178] and stimulating the host immune response [179,180].

One of the first studies to investigate the use of probiotics was conducted over 10 years ago in mice. The authors demonstrated that a course of a common brand of probiotics called VSL#3, which included *Streptococcus thermophiles*, *Lactobacillus* (*species: acidophilus*, *delbrueckii*, *casei* and *plantarum*) and *Bifidobacterium* (*species: breve*, *longum and infantis*) over four weeks was as effective as an anti-TNF antibody at improving liver histology, reducing hepatic lipids, and reducing serum alanine aminotransferase [181]. Importantly, the authors also reported a reduction in pro-inflammatory cytokines and hepatic insulin resistance resulting from modulation of the gut microbiota, although they failed to assess this. This early study into probiotics demonstrates potential for ameliorating multiple hits that are associated with the pathophysiology and development of NAFLD [69,91]. Subsequent to this pioneering study, further animal work has reported that probiotics are effective at reducing cholesterol, low density lipoproteins (LDL), very low density lipoproteins (VLDL), triglycerides [182,183], fat depots [184], hepatic lipid content [185,186,187], steatotic and peroxidase factors and liver aminotransferase [188,189]. There is also evidence demonstrating improvements in hepatic insulin resistance and metabolic control [81,186,190,191]

There is also a body of evidence that has reported reduced inflammation and endotoxemia following probiotic administration period. An exaggerated and damaging inflammatory response occurs in a range of conditions and current evidence associates dysbiosis of the gut microbiota with inflammation, although it is unclear if this is cause or effect. This is especially prevalent in the case of the mucosa and tight protein junctions, where pathogenic bacteria cause damage and increase gut permeability leading to chronic inflammation and endotoxemia [155,192]. Direct modulation of the gut microbiota with viable organisms in probiotics has been shown to reduce hepatic inflammation [188], circulating inflammatory markers [181,191,193], endotoxemia in portal blood [194] and provide essential nutrients for maintaining intestinal epithelium integrity [178,195].

Although these data do imply that probiotics may play a role in the therapeutic management of NAFLD, human data are lacking. In obese children who were non-compliant to lifestyle interventions, probiotics significantly reduced alanine aminotransferase (ALT) and anti-peptidoglycan-polysaccharide antibodies, but did not reduce liver fat [196]. In three well-designed randomized controlled studies, the authors observed that probiotics high in *Lactobacillus gasseri* reduced abdominal adiposity and serum lipids [197,198,199]. However, the duration of these studies was relatively short and the effects on liver steatosis and specific bacterial changes were not reported.

Similar results have been shown in patients with liver disease, where probiotics have been shown to be effective at restoring neutrophil phagocytic capacity in cirrhosis and reducing IL-10, IL-6 and TNF-α secretion and toll like receptor expression [200,201]. More specifically, in NAFLD, the administration of probiotics resulted in a significant reduction liver aminotransferase [202,203], although no changes in hepatic lipids, liver histology or gut microbiota were reported.

The majority of the studies discussed suggest that gut microbiota modulation following consumption of probiotics was responsible for the beneficial effects observed. Early animal data from Cani, Neyrinck, Fava, Knauf, Burcelin, Tuohy, Gibson and Delzenne [81] demonstrated that supplementing mice on a high fat diet with *Bifidobacterium* restored the levels of *Bifidobacterium* comparable to controls on a normal chow fed diet. More recently Bifidobacteria added to animal feed has been shown to increase the abundance of *Bifidobacterium* and *Clostridiaceae* and reduce the abundance of *Enterobacteria* and Eubacteriaceae [184,187,191,193,204]. The changes in bacterial diversity discussed here were also supported by reductions in inflammatory cytokines, endotoxemia, hepatic lipids and gut permeability.

Although existing data suggest probiotics represent a safe and effective treatment option for NAFLD, there are instances where probiotics were ineffective, such as in Crohn’s disease [205] and Helicobacter infections [206]. Such results may simply represent inefficiency of the probiotics selected for these studies and it is plausible different probiotic combinations may yield different results. It should be noted that probiotics are regarded as safe, with little data showing any adverse effects of supplementation. As each disease is uniquely complex, so too must the probiotic selected for treatment, with better characterization of the disease mechanism informing the specific probiotics to use [62]. Important considerations also include the route of administration, dosage, how often to take the treatment, and for how long. As we better understand the most effective means of administering probiotics as well as which specific combinations of bacteria to use, the efficacy of treatment should become apparent. In the era of personalized medicine it is feasible that each NAFLD patient can have their gut microbiota profile determined, allowing probiotics to be tailored to the individual.

## 12. Exercise

Exercise is well recognized for its health benefits and its ability to attenuate the risk of CVD, obesity, mental disorders, diabetes and intestinal diseases [207,208]. More specifically exercise has been shown to be effective at modulating hepatic steatosis and its mediators, improve body composition, liver and adipose tissue insulin sensitivity independent of weight loss [29,47,48,49,209]. Furthermore exercise (both endurance and high intensity) training has been shown to attenuate inflammation and improve insulin secretion by upregulating glucagon-like peptide-1 secretion in the GI tract and pancreas [209,210,211]. Despite the strong associations among exercise, liver health, metabolic control and inflammation, evidence linking exercise, the gut microbiota and NAFLD in humans is lacking. Understanding the interplay between the triad and resulting mechanisms in NAFLD will be fundamental to translating therapeutics into clinical practice.

In a recent study, Clarke, *et al.* [212] investigated the effects of effects of exercise in rugby players compared sedentary overweight and obese controls. The authors observed that the highly active rugby players had a significantly more diverse gut microbiota and lower levels of inflammatory and metabolic markers compared to the controls. Specifically, the authors identified increased relative abundance of Firmicutes, Proteobacteria and reduced relative abundance of Bacteroidetes. These observations are based on extremities of a population with vastly different diet and calorie consumption, thus linking findings directly to the gut microbiota is challenging [18,213]. The authors acknowledge these confounding variables, stating future studies must be well designed in an attempt to isolate the effects that exercise may have on the gut microbiota.

Although animal models do not offer a direct comparison with humans, the control over interventions allows an excellent model to develop disease states and may make it easier to tease out the impact that exercise alone may have on the gut microbiota. To date, there are no animal studies looking specifically at exercise and the gut microbiota in an animal model of NAFLD. There are, however, a number of other animal studies that have investigated the effects of exercise on the gut microbiota in type 2 diabetes [214], obesity, CVD [215], high fat intake [216,217] and low activity levels [218], which are all risk factors for the development and progression of NAFLD [5,6], summarized in Table 5.

The first animal study to investigate the effects of exercise on the gut microbiota was performed nearly a decade ago using rats who voluntarily exercised for five weeks [219]. Rats that exercised had a distinctly different bacterial cluster from the sedentary rats, with a significant increase in bacterial producing bacterium (SM7/11 and T2-87). The exercised mice also consumed fewer calories, had reduced body weight and an in increase in butyrate. Both voluntary and forced exercise has since been shown to elicit significant clustering and increased richness of the gut microbiota, with distinctive changes in the abundance of genus *Lactobacillus*, *Bifidobacterium*, *Dorea*, *Turicibacter*, *Anaerotruncus* and species *Enterococcus faecium* when compared with sedentary animals [220,221,222].

The role of genetic and epigenetic predisposition is unclear, but the gut microbiota evolves with the host from birth [8,12]. Therefore, early manipulation of the microbiota may have beneficial effects later in life. Genetically altered rats with low activity levels from birth had a greater shift in bacterial diversity when compare to the highly active rats [218]. Furthermore, this extenuated increase in bacterial diversity in the low activity rats was supported by a greater improvement in body composition and serum lipid profile, when compared with the highly active mice. The beneficial effects of exercise early in life suggests that even in those with a genetic predisposition to be sedentary may be able to modulate their gut microbiota and risk factors for NAFLD. Further evidence was presented when exercising juvenile and adults rats [223]. Juvenile rats had greater shifts in bacterial composition when compared with the exercising adult rats, which were closely related to body composition of the rats. These studies together suggest that early stimulus and the activity predisposition (low *vs.* high) may be involved in characterizing gut microbiota phenotypes.

Further exercise studies have investigated the effects of exercise on the gut microbiota in hypertension, obesity and diabetes, which are all closely associated with NAFLD [6]. Petriz, Castro, Almeida, Gomes, Fernandes, Kruger, Pereira and Franco [215] exercised obese and hypertensive rats five times per week for four weeks and observed altered composition and diversity of the gut microbiota, with specific increases in *Allobaculum* in hypertensive rats, and *Pseudomonas* and *Lactobacillus* in the obese rats. In a similar exercise intervention, Lambert, Myslicki, Bomhof, Belke, Shearer and Reimer [214] exercised diabetic and control mice and compared them with matched sedentary controls. The authors observed a significant increase in the abundance of several Firmicutes species and reductions in the abundance of *Bacteroides* spp., which had previously been reported in humans [212]. The only human study to look at the acute effects of exercise on gut microbiota was performed in patients with myalgic encephalomyelitis/chronic fatigue syndrome compared to healthy controls [224]. The authors reported that following a single maximal exercise bout the gut microbiota of patients was significantly altered in comparison with controls. Furthermore the patients had a significantly larger level of bacteria recovered in the blood when compared with the healthy controls. Although the authors only conducted a single bout of exercise, they suggest that the altered gut microbiota led to an increase in bacterial translocation and may contribute to worsening myalgic encephalomyelitis/chronic fatigue syndrome. The increased bacteria in the blood may be due to an increase in inflammatory cytokines (IL-6, IL-8, IL-1β, and TNF-α), which have been shown to be required to initiate villus injury and reduce intestinal barrier function [210]. However, the authors failed to report on inflammation, and it must be pointed out that the exercise performed was maximal, which would not be routinely performed. Despite this there is a large body of evidence demonstrating that exercise is able to reduce inflammation [211], hepatic lipids [29], and improves metabolic control [47,209,225,226]. However, further work would need to compare different modalities of exercise and intensities to assess their impact on the gut microbiota, liver fat, metabolic control, inflammation and patients health.

Inter-study variability has been reported by Petriz, Castro, Almeida, Gomes, Fernandes, Kruger, Pereira and Franco [215], who reported increased relative abundance of *Allobaculum*, *Pseudomonas* and *Lactobacillus*. However, Liu, Park, Holscher, Padilla, Scroggins, Welly, Britton, Koch, Vieira-Potter and Swanson [218] reported increased relative abundance of Christensenellaceae, Helicobacteraceae and Desulfovibrionaccae, and Choi, Eum, Rampersaud, Daunert, Abreu and Toborek [221] reported increased relative abundance of the family Enterococcaceae and decreased relative abundance of Erysipelotrichaceae.

Possible reasons for discrepancy reported between studies may include; varying disease type and status amongst studies, exercise intervention duration and/or intensity, diet incorporated (ranging from high fat diet to regular animal chow) and body composition changes. Of particular note, the varying methods and technologies used to extract and sequence the 16S rRNA gene creates potential sources of bias between studies.

Exercise does appear to be able to modulate the gut microbiota and reduce the risk of NAFLD, however, the mechanism(s) remain unknown. Potential mechanisms include: (1) increased butyrate production, which is linked with colonic epithelial cell proliferation and modulation of mucosal immunity and exclusion of pathogens [215,219,227]; (2) increased primary bile acid secretion and cholesterol turnover [228]; (3) growth of beneficial bacteria [221]; and (4) increased core body temperature and reduced blood flow to the GI system reducing gut transit time and substrate delivery to the microbiota [218,229,230]. Although the exact mechanism remains elusive and methodological bias hinders direct cross-study comparisons, existing data indicate that exercise may be able to modulate the composition, diversity and relative abundance of the gut microbiota in NAFLD patients. Further investigation of the impact of exercise on gut microbiota is required and may address why some patients respond to exercise and some do not.

## 13. Conclusions

The gut microbiota has been studied for decades, however, recent developments in 16S rRNA gene sequencing, coupled to advances in computational processing of data has enhanced our understanding of the microbial–host interactions [23]. The gut microbiota has been associated with a range of diseases, from obesity, metabolic syndrome, diabetes, and cardiovascular disease [18,24,25,26] to NAFLD [7,34,36,37,38,39,40,41]. However, existing studies are largely focused on profiling the bacterial community and fail to provide functional information between the gut microbiota and the host. Ultimately, it still remains unknown whether the gut microbiome is *causing* the disease, or simply an *effect* of disease pathophysiology.

This review reveals that diet, pre/probiotics, and exercise play a significant role in the function and diversity of the gut microflora. To date, studies have predominantly focused on pre-clinical models, which have limitations in the transferability of their data to humans. Although much is known, significant questions about how lifestyle therapies may influence the gut microbiota as a therapeutic target for NAFLD care. However, the links between the gut microbiota and NAFLD should continue to be explored to:

(1) better understand inter-patient variability;
(2) develop potential biomarkers for NAFLD development and progression;
(3) understand the mechanism(s) linking the gut microbiota and NAFLD;
(4) develop an understanding of how aspects of lifestyle interventions interact with the gut microbiota and how this may impact upon health; and
(5) tailor prebiotics and probiotics to influence health for each individual.

Furthermore, although these lifestyle interventions clearly impact upon NAFLD, understanding of how they interact with the gut microbiota and NAFLD is lacking and requires longitudinal studies with large sample sizes. For example, the diet has been shown to modulate the gut microbiota in days [62], but these changes are generally reversed in a similar time frame. Therefore, we need to understand the best mechanisms for modulating the long-term establishment of a healthy gut microbiota and the resulting health implications this may have.

As technologies are increasingly developed and the associated costs are reduced, there is huge potential to systematically determine the importance of both the presence of certain bacteria and their ultimate function is a given community. For understanding such complex processes and interaction at the microbe and host levels, there is a need to integrate multiple techniques in a systems biology approach. A focus on large-scale collaborative studies that explore many relevant biological samples to comprehensively determine disease mechanisms and therapeutic efficiency is necessary. This represents an important time for life sciences and the prospect of advances in diseases such as NAFLD is promising.

## Figures and Tables

**Table 1:** Significant bacterial changes following dietary manipulation in the presence of high fat intake. XIV; fourteen, ↑ and ↓ denote increase or decrease in variable, respectively.

Intervention/Treatment	Model Used	Non-Microbiome Changes	Bacterial Changes	Reference
Polyunsaturated fatty acids	Cells	Inhibit growth of mucus	↑ *Lactobacillus casei*	[99]
Oleic acid and *n*-3 fatty acids (EPA and DHA)	Mice	↓ Body Weight	↑ *Clostridial* cluster XIV	[100]
			↑ Enterobacteriales	
			↑ Firmicutes	
			↓ *Bifidobacterium*	
Arabinoxylans	Mice	Improved gut barrier function	↑ *Prevotella* spp.	[102]
		↓ Circulating inflammatory markers	↑ *Roseburia* spp.	
		↓ Adipocyte size	↑ *Bifidobacterium*	
		↓ Body weight gain		
		↓ Serum cholesterol		
		↓ Hepatic cholesterol		
		↓ Insulin resistance		
Chitin-Glucan	Mice	↓ Body weight gain	↑ *Clostridial* cluster XIV	[103]
		↓ Fat Mass		
		↓ Fasting Glucose		
		↓ Hepatic Lipids		
		↓ Cholesterol		

**Table 2:** Significant bacterial changes following high protein intake (↑ and ↓ denote increase or decrease in variable, respectively).

Intervention/Treatment	Model Used	Non-Microbiome Changes	Bacterial Changes	Reference
High Protein/Moderate Carbohydrates High Protein/Low Carbohydrates	Obese Men	↑ Branch chain amino acids	↓ *Roseburia*	[137]
		↑ Phenylacetic acid	↓ *Eubacterium*	
		↑ *N*-nitroso compounds		
		↓ Butyrate		
		↓ Phenolic acids		
High Protein/Low Carbohydrates	Kittens		↑ *Clostridium*	[140]
			↑ *Faecalibacterium*	
			↑ *Ruminococcus*	
			↑ *Blautia*	
			↑ *Eubacterium*	
		
High Protein	Piglets	↑ Branch chain amino acids	↓ *Faecalibacterium* *prausnitzii*	[141]
		↑ Colonic Permeability		
		↑ Cytokine Secretion		

**Table 3:** Significant bacterial changes following prebiotic consumption (↑ and ↓ denote increase or decrease in variable, respectively).

Intervention/Treatment	Model Used	Non-Microbiome Changes	Bacterial Changes	Reference
Prebiotic Diet	Mice	Improved Glucose Tolerance	↓ Firmicutes	[162]
		Improved Leptin Sensitivity	↑ Bacteroidetes	
		↑ GLP-1	Changed 102 taxa	
		↑ L-cell GLP-1		
		↓ Fat Mass		
		↓ Oxidative Stress		
		↓ Inflammation		
Prebiotics—Xylo-oligosaccharide and inulin	Human	↑ Butyrate	↑ *Bifidobacterium*	[164]
		↑ Propionate		
		↓ Acetate		
		↓ P-creso		
		↓ Lipopolysaccharides		
Prebiotics—β2-1 Fructans	Human		↑ *Bifidobacterium*	[172]
Prebiotic—Galactooligosaccharides (GOSs)	Human	↑ Phagocytosis	↑ *Bifidobacterium*	[173]
		↑ Natural killer cells		
		↓ Inflammation		
Prebiotic—Galactooligosaccharides (GOSs)	Human	↓ Inflammation	↑ *Bifidobacterium*	[174]
		↓ IgA		
		↓ Calcoprotectin		
		↓ Cholesterol		
		↓ Insulin		
Prebiotics—Inulin type fructans	Human	↓ Fat Mass	↑ *Bifidobacterium*	[175]
		↓ Plasma Lactate	↑ *Faecalibacterium* *prausnitzi*	
		↓ Phosphatidylcholine	↓ *Bacteroides intestinalis*	
			↓ *Bacteroides vulgatus*	
			↓ *Propionibacterium*	

**Table 4:** Significant bacterial changes following probiotic consumption (↑ and ↓ denote increase or decrease in variable, respectively).

Intervention/Treatment	Model Used	Non-Microbiome Changes	Bacterial Changes	Reference
Probiotic—oligofructose and Bifidobacterium species	Mice	↓ Endotoxemia	↑ *Bifidobacterium*	[81]
		Improved glucose tolerance		
Probiotic—Bifidobacterium longum	Rat	↓ Endotoxemia	↑ *Bifidobacterium*	[185]
		↓ Inflammation		
		↓ Intestinal myeloperoxidase		
		↓ Body Weight		
		↓ Fat Depots		
		↓ Systolic Blood Pressure		
		Improve insulin sensitivity		
Probiotic—Bifidobacterium longum or Lactobacillus acidophilus	Rat	↓ Hepatic Lipids	↑ *Bifidobacterium longum*	[188]
			↑ *Lactobacillus acidophilus*	
Probiotic—Bifidobacterium pseudocatenulatum	Mice	↓ Cholesterol	↑ *Bifidobacterium*	[192]
		↓ Triglycerides	↓ Enterobacteria	
		↓ Glucose levels		
		↓ Insulin resistance		
		↓ Leptin		
		↓ Inflammation		
		↓ Hepatic Lipids		
Probiotic—Bifidobacterium pseudocatenulatum	Mice	↓ Inflammation	↓ *Firmicutes*	[194]
		↓ Endotoxemia	↓ *Proteobacteria*	
		↓ B cells		
		↓ Macrophages		
		↓ Cholesterol		
		↓ Body Weight Gain		
		↓ Triglycerides		
		↓ Insulin resistance		
Probiotic—Bifidobacterium breve	Mice	↑ Propionate	↑ *Clostridiaceae*	[205]
			↓ *Eubacteriaceae*	

**Table 5:** Significant bacterial changes following exercise (↑ and ↓ denote increase or decrease in variable, respectively).

Intervention/Treatment	Model Used	Non-Microbiome Changes	Bacterial Changes	Reference
Controlled treadmill running	Mice		↑ *Lactobacillus* spp.	[215]
			↑ *Clostridium leptum* (C-IV)	
			↓ *Clostridium* cluster (C-XI)	
			↓ *Bifidobacterium* spp	
Controlled treadmill running	Rat	↓ Blood Lactate	↑ *Allocaculum*	[216]
			↑ *Pseudomonas*	
			↑ *Lactobacillus*	
			↓ *Streptococcus*	
			↓ *Aggregatibacter*	
			↓ *Sutturella*	
Voluntary wheel running	Mice	↓ Body Weight	↑ Bacteroidetes	[217]
		↓ Body Fat	↓ Firmicutes	
		↓ Blood glucose	↓ Actinobacteria	
		↑ Heart:Body Weight		
Controlled wheel running	Mice		↓ *Streptococcus*	[218]
			↓ Bacteroidetes	
			↑ Firmicutes	
Voluntary wheel running	Rat	↓ Body Fat	↓ Firmicutes	[219]
		↑ Lean Body Mass	↑ Cyanobacteria	
		↓ Non-esterified fatty acids	↑ Proteobacteia	
		↓ Cholesterol		
Voluntary wheel running	Rat	↑ Cecal size and weight	↑ SM7/11	[220]
		↑ Butyrate production	↑ T2-87	
		↓ Body Weight		
Voluntary and forced treadmill running	Mice	↓ Body Weight	↑ *Dorea*	[221]
			↑ *Anaerotruncus*	
			↑ *Nautilia*	
			↑ *Coprococcus*	
			↑ *Oscillospira*	
			↓ *Turicibacter*	
			↓ *Moryella*	
			↓ *Prevotella*	
Voluntary wheel running	Mice	↓ Body Weight	↑ Enterococcsceae	[222]
			↑ Staphylococcsceae	
			↓ Erysipelotrichaceae	
Voluntary wheel running	Rat	↑ Body Weight	↑ *B. Coccoides-E Rectale*	[223]
		↑ Serum Leptin	↑ *Lactobacillus*	
		↓ Serum Ghrelin	↓ *Clostridium*	
			↓ *Enteroccocus*	
			↓ *Prevotella*	
			↓ *Bacteroides*	
Voluntary wheel running	Rat	↑ Body Weight	↓ *Rikenellaceae* g_AF12	[224]
		↑ Lean Body Mass	↓ *Rikenellaceae* *g*	
			↓ *Desulfovibrio spp*	
			↑ *Blautia* spp	
			↑ *Turicibacter*	
			↑ *Anaerostipes spp*	
			↑ *Methanosphaera*	
Single Peak Exercise Test	Human	↑ Bacteria in blood	↑ Actinobacteria	[225]
			↑ Firmicutes	
